# Mapping the global public health intelligence landscape: a multiregional cross-sectional survey

**DOI:** 10.1186/s12889-025-25406-0

**Published:** 2025-12-23

**Authors:** Alba Méndez-Brito, Romy Kerber, Gianfranco Spiteri, Kyeng Mercy Tetuh, Angela Fehr, Christine Manthey, Kokou Nouwame Alinon, Sarah Esquevin, Neil Squires, Andreas Jansen, Carlos L. Correa-Martínez

**Affiliations:** 1 Department of Infectious Disease Epidemiology, Robert Koch Institute, Nordufer 20, Berlin, 13353 Germany; 2 Postgraduate Training for Applied Epidemiology (PAE), Department of Infectious Disease Epidemiology, Robert Koch Institute, Berlin, Germany; 3 ECDC Fellowship Programme, Field Epidemiology path (EPIET), European Centre for Disease Prevention and Control (ECDC), Stockholm, Sweden; 4 Public Health Functions Unit, European Centre for Disease Prevention and Control, Stockholm, Sweden; 5 Division of Surveillance and Disease Intelligence, Africa Centres for Disease Control and Prevention, Addis Ababa, Ethiopia; 6 Centre for International Health Protection, Robert Koch Institute, Berlin, Germany; 7 EpiStack, Atlanta, GA USA; 8 International Association of National Public Health Institutes (IANPHI), Berlin, Germany

**Keywords:** Public health intelligence, Epidemic intelligence, Disease surveillance, Early detection, Public health threats, Surveillance systems, Indicator-based surveillance, Event-based surveillance

## Abstract

**Background:**

Public health intelligence (PHI) allows for timely detection of public health threats. Exchange and close cooperation between PHI teams are crucial for early threat detection and standards harmonization, yet a comprehensive overview documenting their activities is lacking. We aimed to enhance mutual awareness and collaboration possibilities by mapping and characterizing PHI teams.

**Methods:**

We developed and distributed an online survey through network sampling (June-November 2023) via regional Centres for Disease Prevention and Control (Africa CDC, ECDC, US CDC), the International Association of National Public Health Institutes (IANPHI), the Robert Koch-Institute, and the World Health Organization (WHO). We identified and described PHI teams and their activities, including implementation of PHI processes, workforce, capacity building, priorities and perspectives on challenges and opportunities, by WHO regions and type of institution.

**Results:**

We identified 132 PHI teams from 87 countries in all regions, primarily in public health institutions (40%, 53/132) and ministries (27%, 35/132). Potential public health threats are monitored at national (39%, 51/132), international (20%, 26/132), and both levels (42%, 55/132). Most teams (89%, 114/128) integrate indicator-based and event-based surveillance. Teams focus mainly on human communicable diseases (95%, 125/132), healthcare-associated infections/antimicrobial resistance (66%, 87/132) and health in natural disasters (64%, 85/132). Nearly half of the teams (43%, 50/117) are not part of early event detection networks. All teams based in regional public health institutions (7/7) and United Nations (UN) institutions (10/10) are able to scale up their activities if needed. More than half of the teams (59%, 71/120) provide training activities on early detection. There is a strong interest in capacity building and networking.

**Conclusion:**

PHI teams worldwide perform partially overlapping tasks, suggesting benefits from broader exchange. The interest in trainings and networking underscores the need for platforms supporting exchange, peer-to-peer cooperation and capacity building. International partner institutions are key in fostering global development of PHI.

**Supplementary Information:**

The online version contains supplementary material available at 10.1186/s12889-025-25406-0.

## Background

In our globalized world, the timely detection of, and response to, public health threats has become imperative, as recently demonstrated by the COVID-19 pandemic and global mpox clade I outbreak. Preparedness for future health emergencies requires robust systems capable of early detection, assessment, and reporting. This relies on the timely connection of event-based surveillance (EBS) and indicator-based surveillance (IBS) data. EBS involves the collection and analysis of unstructured information (for example, hotlines), while IBS relies on the systematic data collection from traditional epidemiological indicators (for example, sentinel surveillance of influenza) [1]. The connection of IBS and EBS focused only on infectious diseases is referred to as epidemic intelligence (EI), while public health intelligence (PHI) encompasses additionally non-infectious diseases and further threats [2]. PHI is defined by the WHO as a core public health function responsible for identifying, collecting, connecting, synthesizing, analyzing, assessing, interpreting, and generating a wide range of information for actionable insights, and disseminating these for informed and effective decision-making to protect and improve population health [3].

Although PHI is a well-defined concept and policy documents consistently call for strengthened PHI activities to ensure better preparedness and response [4], there is a notable lack of a comprehensive overview on global PHI activities, despite some evidence being available for specific regions or institutions [5, 6]. The absence of an overview hampers the standardization of procedures and quality assurance, the development of uniform training programs, the establishment of robust networks, effective data sharing, and technical exchange. An overview of the current PHI landscape is essential for better coordinated and effective PHI practices to improve global health security. We therefore performed a comprehensive mapping and characterization of PHI teams globally, aimed at increasing mutual awareness and cooperation, describing PHI practices, challenges and opportunities.

## Methods

We conducted a global online cross-sectional survey to identify and describe teams performing PHI activities. We developed a semi-structured questionnaire in English, translated it into French, Russian and Spanish and piloted it internally in each language for refinement. Efforts were directed towards identifying as many teams performing PHI activities as possible. Therefore, we employed both network and snowball sampling methods [7]. The survey was distributed between June 2023 and November 2023 through the established networks of the Africa Centres for Disease Prevention and Control (Africa CDC), the European Centre for Disease Prevention and Control (ECDC), the International Association of National Public Health Institutes (IANPHI), the Robert Koch Institute (RKI), the United States Centers for Disease Control and Prevention (US CDC), and the Epidemic Intelligence from Open Sources (EIOS) Core Team at the WHO Hub for Pandemic and Epidemic Intelligence (network sampling). Hence, mostly teams with a focus on human health were contacted. Recipients were instructed to complete only one survey per team and invited to share the link to the questionnaire with other teams performing similar activities within their own networks (snowball sampling).

The survey targeted teams engaged in PHI activities across various institutions worldwide with national and supranational coverage. To accommodate the diverse terminology, we defined our focus as “teams performing activities for the early detection of public health threats,” encompassing PHI and EI and providing definitions of the terms.

The survey consisted of 36 open-ended and closed questions (Table S1) in 5 sections (Table 1). Respondents were informed of the study’s objectives and had to provide consent prior to starting the survey. They had the option to skip questions if they chose not to answer, and they could withdraw from the survey at any time while still permitting the use of their partially completed responses. Therefore, there are small variations in the denominator for results of single answers along the survey. Open-ended questions were analyzed through thematic analysis, which was conducted by a single researcher without employing a predefined framework. Responses were systematically reviewed in Excel to identify broad categories, which were subsequently grouped into overarching themes or categories.

**Table 1:** Global public health intelligence landscape survey structure by sections, including description of the section, topics covered and question numbers

	Description	Topics covered	Question number
Section 1	General information	Institution, country, self-reported performance of early detection activities, mandate, date and reason of establishment	1–6
Section 2	Implementation of PHI activities	Geographic scope, hazards monitored, surveillance activities and their frequency, methods for data collection, reporting findings, upscaling activities, detailed assessments, collaboration	7–19
Section 3	Workforce and capacity building	Dedicated resources, staff background and requirements, training provided to staff, training capacities and interest	20–28
Section 4	Perception and opinion questions	Perceived priorities, threats and opportunities	29–31
Section 5	Other questions, including networking	Future of activities, current networking and networking interest, survey reception and other remarks	32–36

We described the teams’ implementation of PHI processes, workforce, capacity building, priorities and perspectives on challenges and opportunities, by WHO regions and type of institution, using frequencies and proportions.

Duplicate responses —defined as multiple surveys filled in by the same team— were identified and excluded from further analysis according to predefined criteria (Figure S1). The survey was implemented using the Voxco software; data analysis was conducted using the R software. Approval for data collection was obtained from the data protection officer at the Robert Koch Institute.

## Results

### General information

In total, 132 responses from 261 questionnaires received were included. Responses were excluded due to early survey dropout, unwillingness to participate, the team not performing PHI activities, or duplicate responses (see flowchart in Figure S2). All distributing partner institutions successfully engaged participants. Specifically, 32% (37/114) received the survey from RKI, 25% (29/114) through Africa CDC, 16% (18/114) through the EIOS community, 14% (16/114) from ECDC, 15% (17/114 teams) via IANPHI, and 7% (8/114) from US CDC. Additionally, 4% (5/114) received the survey from colleagues within their own institutions, and 11% (13/114) through other channels.

**Fig. 1:**
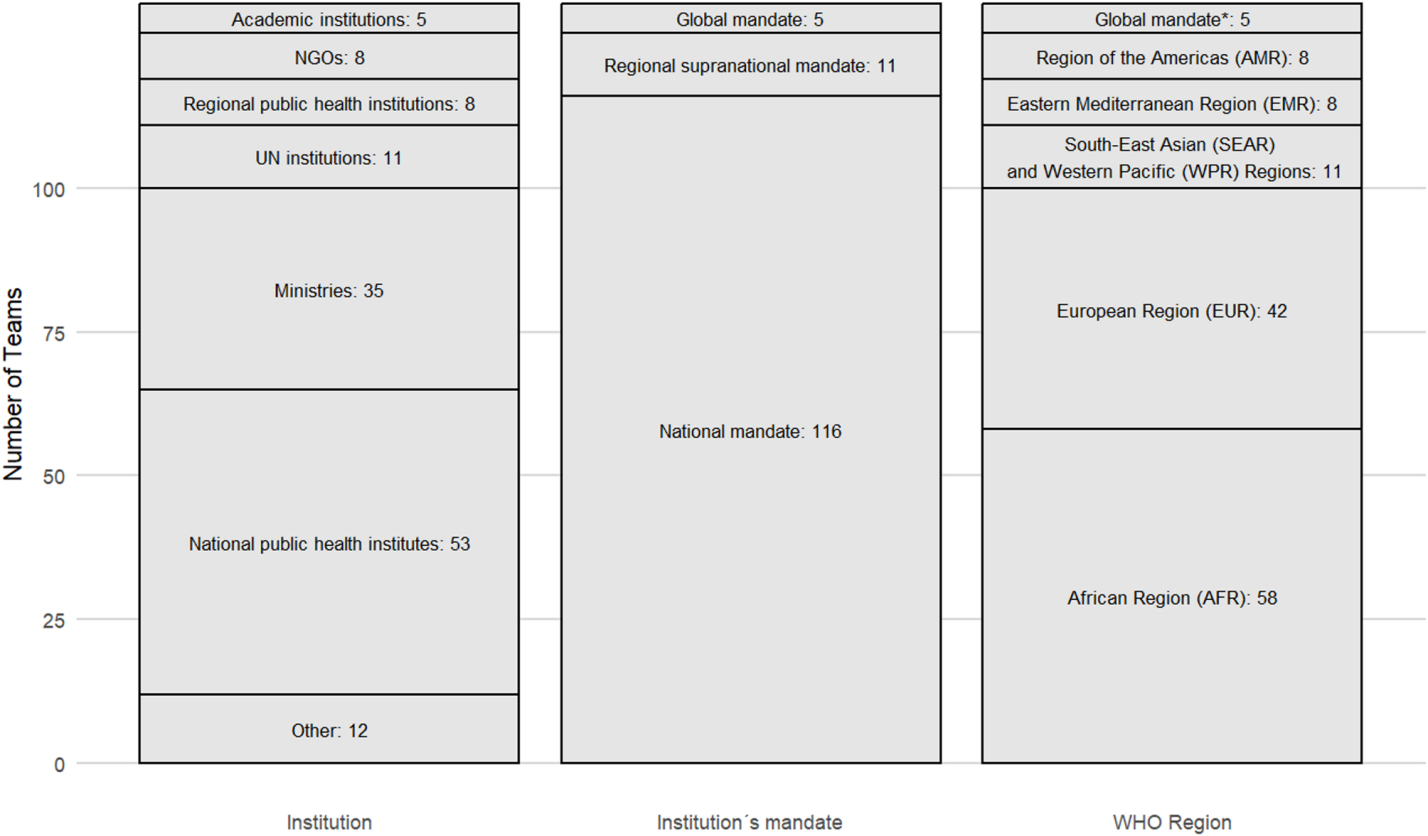
Description of the included teams in the global public health intelligence landscape survey by institution, institutional mandate and World Health Organization (WHO) Region (*N* = 132). * Institutions with global mandates are not assigned to a specific WHO Region, as their scope spans worldwide. AMR = Region of the Americas, AFR = African Region, EMR = Eastern Mediterranean Region, EUR = European Region, SEAR and WPR = South-East Asia and Western Pacific Regions

**Fig. 2:**
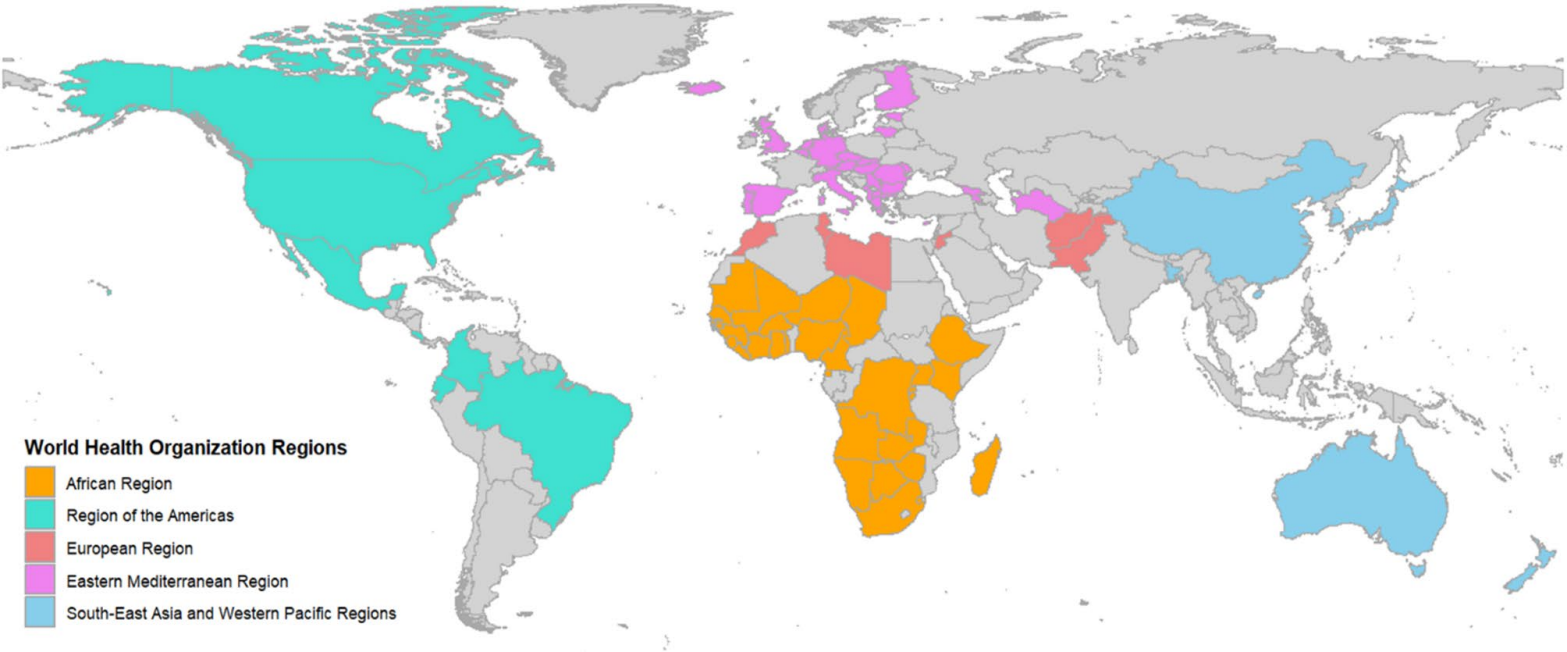
Map of respondent countries in the global public health intelligence landscape survey. Teams within institutions with specifically global or regional supranational mandate are not included (*N* = 87). This map does not imply the expression of any opinion by the authors or their institutions regarding the legal status of any country, territory, city, or area, or of its authorities, nor does it imply any opinion on the delimitation of frontiers or boundaries

The teams are situated most commonly within national public health institutes (NPHIs), ministries, UN institutions, NGOs, academic institutions, and regional public health institutions (RPHIs), which include both regional public health agencies such as Africa CDC and ECDC, as well as further actors with a regional mandate (Fig. 1). Regarding their institutions’ mandate, 88% of teams (116/132) were located in an institution with a mainly national mandate, such as national public health institutes or ministries, 8% (11/132) in institutions with a regional supranational mandate, such as regional public health institutions, and 4% (5/132) in institutions with a global mandate, such as UN institutions or international NGOs. In total, 116 out of 132 teams were located in institutions with a national mandate, representing 87 countries from all WHO regions: African Region (AFR), Region of the Americas (AMR), European Region (EUR), Eastern Mediterranean Region (EMR), South-East Asian Region (SEAR), and Western Pacific Region (WPR) (Figs. 1 and 2). Institutions with supranational or global mandates are not assigned to specific countries in our analyses, as their scope spans multiple countries or worldwide. Since there is only one respondent country from SEAR, these results are grouped with those of WPR under consideration of the geographical context. A list of respondent countries can be found in Table S2. In most cases, one to two teams per country were identified, respectively 84% (97/116) and 14% (16/116). In total, 83% of the teams (109/132) were established prior to the COVID-19 pandemic. In 44% (45/132) of the cases teams were created as a result of specific health threats (e.g. Ebola outbreak in West Africa in 2013, COVID-19 pandemic, influenza events, cholera outbreaks, SARS outbreak in 2002).

### Implementation of PHI activities

Activities reported by teams included, among others, informing response activities, providing information for risk assessments and informing policy makers (Table 2). In total, 39% (51/132) of the teams monitor events only at national, 20% (26/132) only at international and 42% (55/132) at both levels. Table 2 contains an overview of the type of threats monitored, with details by WHO Region provided in Table S3. A One Health approach (considered as the monitoring of human, animal and environmental health events) is followed by 43% (57/132) of the teams, specifically 59% (34/58) in AFR and 24% (10/42) in EUR. An all-hazards approach (considered as the monitoring of chemical and nuclear hazards, besides biological ones) was followed by 31% (41/132) of teams identified.

**Table 2:** Counts and percentages of respondent teams in the global public health intelligence landscape survey, by mandate, monitored hazards, information sources, and methods used

		Nr of teams	Percentage
Mandate		**N=132**	
	Inform action and response activities	120	91%
	Provide information for risk assessments	111	84%
	Inform policy makers	109	83%
	Inform the public	92	70%
	Inform the scientific community	89	67%
	Generate scientific evidence for research purposes	67	51%
Monitored hazards		**N=132**	
	Human health: communicable diseases	125	95%
	Healthcare-associated infections or antimicrobial resistance	87	66%
	Health in natural disasters	85	64%
	Food safety and security	81	61%
	Environmental health	78	59%
	Health in vulnerable populations	76	58%
	Animal health	73	55%
	Chemical hazards	61	46%
	Human health: non-communicable diseases	55	42%
	Nuclear hazards	42	32%
Information sources		**N=132**	
	Routine monitoring of established epi indicators (IBS)	120	95%
	Facility-based detection of relevant events (EBS)	88	66%
	Targeted screening of traditional media (EBS)	88	64%
	Community-based detection of relevant events (EBS)	84	61%
	Expert networks (EBS)	72	59%
	Targeted social media scanning (EBS)	70	58%
Methods used		**N=128**	
	Newsletters or mailing lists	77	60%
	Official websites	100	78%
	Moderated web aggregators:		
	ProMED	62	48%
	Outbreak News Today	46	36%
	GPHIN	32	25%
	Other moderated web aggregators	13	10%
	Automatic web aggregators:		
	EIOS	51	40%
	Healthmap	34	26%
	Medsys	18	14%
	Other automatic web aggregators	3	2%
	Public information channels:		
	Call centers/hotlines	57	44%
	SMS	46	36%
	E-mails	70	54%
	Messaging apps (WhatsApp, Telegram, etc.)	21	16%
	Other public information channels	52	40%
	Other methods	11	9%

Specific events are monitored on a regular basis by 84% of teams (108/128). An open-ended question revealed that although the monitoring of events is context dependent, the ones more frequently monitored globally, in descending order of frequency, include various influenza events (avian, pandemic, and seasonal influenza), measles outbreaks, hemorrhagic fevers (such as Ebola, Marburg and Lassa Fever), cholera outbreaks, antimicrobial resistance, malaria, and HIV/AIDS.

The connection of IBS and EBS for PHI varies among teams. Data from EBS sources are combined with data from IBS in the context of PHI by 89% (114/128) of teams. Routine monitoring of epidemiological indicators (IBS) is performed by 95% of the teams (Table 2). Regarding EBS, teams in AFR predominantly rely on information collected through facility-based (84%, 49/58) and community-based surveillance (81%, 47/58). In EUR, information from expert networks (83%, 35/42) and targeted screening of traditional media (76%, 32/42) is predominant. In AMR, EMR, and WPR/SEAR no predominant approaches were observed (Table S3).

Globally, the most frequently employed methods for EBS data collection comprise the use of secondary sources (e.g. official institutional websites, newsletters/mailing lists, e-mails) as well as primary sources, including channels to receive information from the general public, such as call centers/hotlines and public e-mail addresses (Table 2). Regarding specific platforms or tools, ProMed (Program for Monitoring Emerging Diseases) is the most commonly used among respondents, followed by the EIOS platform and Outbreak News Today, among others. In Table S4 we provide an overview of all platforms and tools mentioned.

Daily event monitoring is conducted by 79% (101/128) of teams. Specifically, 58% (74/128) maintain this daily monitoring regimen even during weekends and holidays. Conversely, 10% teams (13/128) conduct monitoring at least once a week, and 5% (6/128) do so less than once a week. Only 6% (8/128) of teams base their monitoring frequency on the occurrence of specific seasonal or unexpected events. Less than three quarters (72%, 41/57) of the identified teams in AFR monitor events during weekends and holidays, this accounts for 75% (7/8) of the teams in AMR and 55% (6/11) in SEAR/WPR. In EUR and EMR, daily routine monitoring of events on weekends is less common, accounting for 41% (18/44) and 38% (3/8) of teams. For nearly all types of institutions, excluding academia, the most common frequency of monitoring is daily, at least on weekdays. In case acute events arise, 78% (96/128) of teams upscale their activities by either increasing the frequency of activities (51%, 65/128), hiring additional staff and extending working hours (51%, 65/128), or both (43%, 51/128). All participating UN institutions (10/10) and RPHIs (7/7) report being able to increase their activities in acute events (Table S5).

A wide majority of teams 91% (113/124) perform detailed analyses and risk assessments of events, with 29% (36/124) doing it for all events and 62% (77/124) only for selected events. Detailed analyses for all detected events are conducted by 57% of RPHIs (4/7) and 50% (4/8) of respondent NGOs. For these detailed analyses, 93% (114/123) of the teams consult with team external experts, including other teams within the same institution, subject matter experts, or professionals from other specialized institutions.

Reports are produced by 96% (123/128) of PHI teams. Among these, 69% (85/123) share reports internally within their own institution, 66% (81/123) also share these with external stakeholders, and 31% (38/123) communicate single pieces of information via e-mail or telephone. Additionally, 25% (31/123) develop regular reports for external, public distribution. RPHIs are the respondent institutions that most commonly share regular reports publicly (43%, 3/7), while none of the teams from academia (0/4) and NGOs (0/8) publish their outputs.

### PHI workforce and capacity building

Of the PHI teams 72% report that their institutions have dedicated human resources specifically for early detection of public health threats (Table S3). In 88% (7/8) of teams based in UN institutions, staff for early detection is permanently available (Table S5).

Respondents were asked to indicate the professional backgrounds represented in their team (i.e. in which areas staff have received academic training) by selecting all that applied from a predefined list and indicating further areas of expertise if necessary. Among the 121 respondent teams, the majority reported having staff with backgrounds in epidemiology (90% of teams), public health (88%) and medicine (84%), followed by nursing (45%), biology (40%), and veterinary medicine (40%). Technical expertise in statistics (53%) and information technology (33%) is also present. Backgrounds in social sciences are the least common.

Specific professional requirements for new staff are in place in 96% (117/122) of the teams. These include having a university degree in a relevant field (71% of the teams, 87/122), having other professional qualifications (65%, 79/122), having previous experience in early detection of public health threats (40%, 49/122), and completing internal or external courses (40%, 49/122). Internal continuous training is offered in 67% of the teams (81/121), being mandatory in 28% (34/121) and optional in 40% (48/121) of the cases.

Among respondents, 44% (53/121) reported to be unaware of any external training programs for early detection of public health threats. Reasons mentioned by respondents in an open-ended question for not participating in trainings are lack of funding, lack of time, and no knowledge of such programs. All respondents (121/121) confirmed that their teams would be willing to receive trainings on early detection of public health threats. Currently, 59% of the teams (71/120) across all WHO regions offer such trainings to other teams. RPHIs (100%, 7/7) and UN bodies (88%, 7/8) are the institutions most frequently providing trainings for early detection of public health threats (Table S3).

### Networking

Some level of cooperation between teams conducting early detection activities in different institutions was reported by 92% of the teams (112/122); either regularly (48%, 59/122) or on an event-related basis (43%, 53/122). For detailed analyses or assessments, 93% of the identified teams (114/123) across all regions and institution types consult with internal or external experts. Only 43% (50/117) of the responding teams are part of a network or community of practice, and none of the teams from academic institutions participate in one. Involvement in national networks (e.g. with ministries of health, agriculture, and environment), as well as participation in networks from UN institutions (e.g. EIOS initiative, the Global Outbreak Alert and Response Network (GOARN), WHO Regional Offices, WHO International Health Regulations (IHR) Focal Points), was reported in an open-ended question. Additionally, regional entities were highlighted for their role in enabling networking, particularly Africa CDC (Regional Coordination Centres and the African Health Volunteers Corps), ECDC (EpiPulse), the European Commission (Early Warning and Response System (EWRS), and the Global Health Security Initiative (Early Alerting and Reporting (EAR)).

In total, 91% (106/117) of the teams confirmed their interest in further networking to exchange experiences (best practices, methods, protocols), information for early detection and response, and capacity building. The practicality of having these networks focused on neighboring countries or those similar in size or resources was identified as important factor for rapid communication and cooperation.

### Perceptions and opinions

**Fig. 3:**
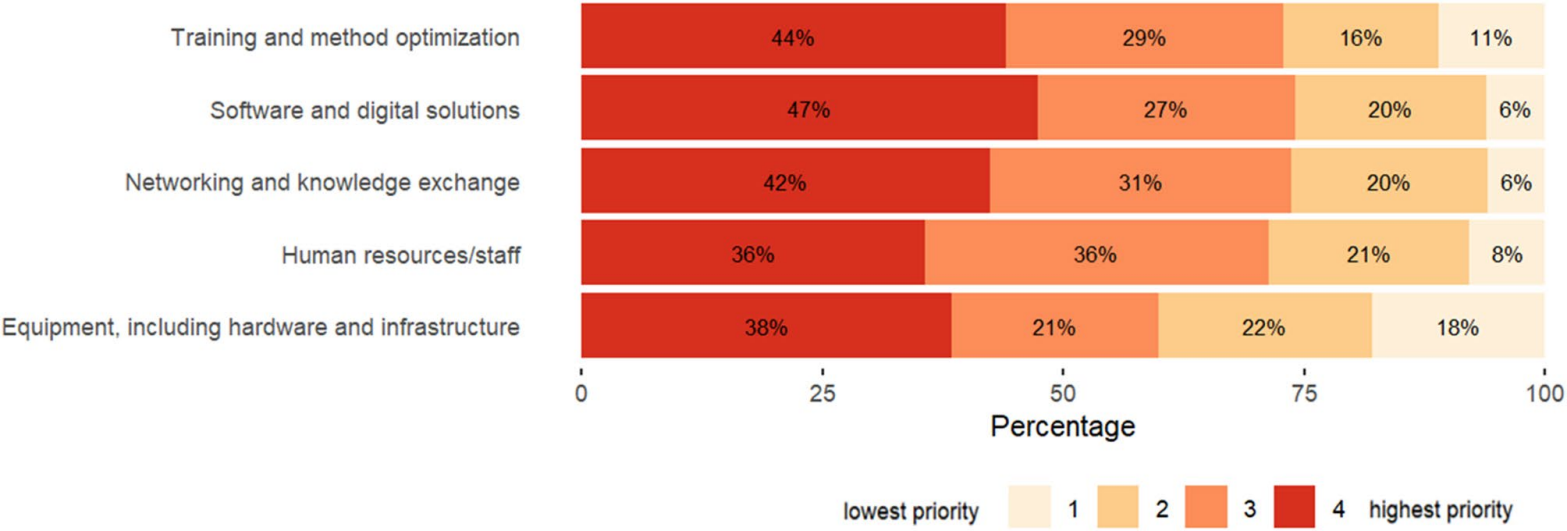
Percentage distribution of teams in the global public health intelligence survey assigning each priority level to predefined categories (1 = lowest, 4 = highest), (*N* = 119)

Respondents were asked to assign a priority level to different predefined categories: training and method optimization, software and digital solutions, networking and knowledge exchange, human resources/staff, and equipment, including hardware and infrastructure (Fig. 3). They could choose between 4 priority levels for each category. All categories were assessed to have the highest priority level by more than 30% (36/119) of the respondent teams. Equipment, including hardware and infrastructure, was considered the least pressing issue, with the lowest percentage of teams assigning it the highest or second-highest priority level. Results varied slightly by region and by type of institution (Figure S3). A higher percentage of respondents from NGOs and academia considered all predefined categories to be at the highest priority level in comparison to other types of institutions.

Several opportunities to strengthen PHI activities were identified by respondents in an open-ended question and thematically grouped into: a) increase funding (domestic and international sources, e.g. for emergency preparedness from the Pandemic Fund); b) increase awareness of public health threats as a key factor for mobilizing resources; c) mobilize political will, commitment and support (e.g. IHR, pandemic accord, national health reforms); d) implement training and capacity building; e) implement technological advancements and digital solutions (machine learning for risk assessment, disease modelling, data and systems integration); f) initiate or strengthen international collaboration and knowledge exchange (national, regional and global networks). 

The following challenges were mentioned in an open-ended question: a) reduced funding e.g. through COVID-19 pandemic waning, which undermines long-term planning and stability of public health programs; b) lack of political will and political choice for preventive prioritization of public health in the absence of pressing threats; c) deficits in human resources, (shortage of qualified staff, high staff turnover, insufficient training opportunities, lack of incentives to attract and retain skilled personnel); d) technological and infrastructure limitations (inadequate IT systems and infrastructure); e) ineffective coordination and communication (nationally and internationally); f) misinformation and disinformation among the public; g) lack of standardized systems for EBS.

Teams reported on perspectives for their work after COVID-19 reaching an endemic phase. While 16% (19/118) could not assess whether this will affect their activities, 36% (43/118) reported that activities will be scaled up. In contrast, 20% (24/118) reported a reduction of activities, and 27% (32/118) reported no planned changes.

## Discussion

This study presents the first comprehensive mapping and characterization of PHI teams globally. We identified 132 teams performing PHI activities across all WHO regions, situated in various types of institutions. The identified teams represent a sample of the global PHI universe obtained through network and snowball sampling.

Nearly half of the teams identified were established following specific public health threats, underscoring how acute public health events can motivate political decision-making and the mobilization of resources to enhance surveillance capabilities. At the same time, it reflects a reactive approach that, as observed following past public health crises, may lack sustainability [8]. Many teams reported this potential lack of long-term sustainability as significant. A quarter of the teams had expected a reduction of their resources and activities after the acute phase of the COVID-19 pandemic came to an end. This can hamper their ability for early detection of events which is necessary to respond to public health risks in a timely manner as mandated by the IHR [9].

The significance of supranational actors in supporting countries is highlighted, as all respondent UN institutions and RPHIs reported having permanent human resources for PHI activities and the capacity to rapidly scale up their operations when needed, whereas countries reported these to a lesser extent. This is relevant not only to face acute public health threats, but also to strengthen national capacities for enhanced country-specific threat detection and assessment. Similarly, UN institutions and RPHIs have significantly more capacity to perform detailed threat analysis and risk assessments than national PHI teams, underscoring the value of outputs and suggesting opportunities for methodological knowledge exchange [10–12]. Given the significant decrease in global health funding since the survey was conducted, the role of funders and the distribution of resources have changed substantially, making it imperative to redefine priorities and funding flows. These developments are likely to impact PHI activities as well. As noted by some authors, such shifts can also create opportunities to build resilience in affected countries, encouraging innovative strategies and a stronger focus on health system priorities [13]. Emphasizing cooperation as a fundamental pillar of PHI, through horizontal, country-to-country collaboration and knowledge exchange, could reduce reliance on vertical funding and help establish resilient systems capable of effective early warning and detection.

A large number of teams monitor similar threats internationally, suggesting overlap in the PHI work. This indicates that resources are being invested in several countries simultaneously with similar purposes. Such overlaps could however be harnessed, providing a valuable starting point for cooperation and synergies among teams. Mutual awareness, interconnection and collaboration within the international PHI community are needed in order to avoid duplication, harmonize event assessments, and come to a more efficient organization. In this context, our findings provide a reference for the hazards and specific diseases commonly monitored by PHI teams, as well as the surveillance types, methods, and tools they employ, offering a basis to strengthen coordination and collaboration across institutions.

Despite more than half of the teams engaging in some level of cooperation with other PHI teams, almost all respondents expressed their interest in further expanding their networks. According to our results, a large proportion of teams consider capacity building, process optimization and digital approaches to be high-priority topics. In light of this interest, these areas could potentially serve as channels for exchange, yielding quick wins in terms of collaboration, activity alignment and technical consistency. RPHIs, WHO, and networks like GOARN and IANPHI could capitalize on this interest in order to foster collaborative surveillance under their mandate, a concept envisioned by the WHO as a key element to combat fragmentation at national, regional, and global levels [14]. Recent developments regarding global health funding calls for these organizations to engage with potential new funders. This should allow to streamline resources for strengthening PHI activities by taking advantage of the ample expertise and platforms offered by existing reference organizations. Some networks already established, such as the regional Communities of Practice from Africa CDC [15], the ECDC EI network [16], and the EIOS Community of Practice [17], are widely considered by the respondents to be beneficial for these purposes and constitute good examples for further networking efforts. Respondents pointed out the need for regional networks that consider local conditions, needs, aims and commonalities among neighboring countries. Notably, academic institutions indicated that they are not actively involved in PHI networks. This points at a gap in knowledge transfer that needs to be addressed in order to maximize the reach and effectiveness of PHI activities, while information security aspects need to be considered. Since many teams are based at national public health institutes, there may also be ample opportunity to cooperate and organize knowledge exchange under the umbrella of IANPHI and its thematic committees [18].

Regional differences were observed in the adoption of One Health approaches. AFR is the region with the highest proportion of teams using a One Health approach in their surveillance activities, as also shown by previous evidence [19]. The importance of One Health for the region has been acknowledged by key actors such as Africa CDC [20, 21]. This proportion is lower in EUR, although evidence suggests that engagement between public health and animal health institutes exists. However, according to previous work, integration, standardization, and automatization of efforts can be improved in the region [22]. This could be achieved, for example, by leveraging the interconnectedness of key institutions involved in human and animal health in the region, such as ECDC, the European Food Safety Authority and the Regional Representation of the World Organization for Animal Health, drawing on their links to countries and reshaping vertical lines of work in favor of cross-institutional, multidisciplinary work to foster regional One Health approaches. Professional profiles of PHI staff in the respondent teams tend to be concentrated in the medical sciences, with comparatively fewer staff with backgrounds such as statistics, information technology, and social sciences, indicating limited interdisciplinarity within many teams. According to a number of experts convened by WHO to reflect on innovation strategies in public health surveillance, interdisciplinarity should be sustainably enhanced to strengthen, among others, practices under the One Health and all-hazards approaches [23]. Considering the results of our survey, this statement could be applied to the PHI teams identified in the survey, which could profit from further expertise in other areas of knowledge in order to enhance threat detection throughout different sectors. It should be noted that this survey was distributed among networks consisting primarily of public health institutions with a mandate focused on human health. A limited outreach among institutions such as animal health agencies or ministries of wildlife and agriculture could have hence led to a partial representation of One Health practices.

Most of the respondent teams connect IBS and EBS data for the early detection of public health threats, with only a few teams focusing exclusively on one. However, the lack of published guidance and recommended procedures for performing PHI, including connection of IBS and EBS data was identified as a challenge. Certain types of EBS are more widely used in some regions than others. For example, healthcare facility-based in AFR plays a predominant role, while media screening is the most widely employed EBS type in EUR according to the respondents. These results should be interpreted considering structural differences in surveillance systems and notification paths, which may lead to certain components (e.g. laboratory-based surveillance) to be more closely integrated into IBS or EBS, depending on local dispositions. While a more detailed differentiation of how this integration occurs in specific surveillance systems warrants further research, our results reveal that strengthening PHI capacities requires tailored strategies based on the needs, priorities and contextual factors in each country or region. Commonly used methods for EBS data collection included reviewing institutional websites and newsletters as well as establishing hotlines and dedicated e-mail addresses for the general population to share relevant information. This highlights the importance of mixed strategies to increase sensitivity of EBS activities in accordance to the context where these are being conducted. While IBS approaches are largely standardized, few documents compiling guidance on implementation of EBS and context adaptation have been produced [1, 24]. Approaches for monitoring and evaluating EBS have been explored in recent years, including the indicator framework created by Africa CDC, which provides an evidence-based method for assessing EBS systems and the evaluation of specific tools, such as EIOS [25, 26]. Sustained efforts to standardize and refine these approaches are needed to improve EBS performance across regions.

Technology and digital solutions are mentioned by a high number of teams as valuable resources to enhance PHI work. This coincides with previous work reporting that professionals in the area of early warning wish to optimize their procedures to manage an increasing amount of data and have methodological support for cross-sectoral analysis [22]. To meet these needs, more effective tools and broader access are essential, allowing for better insights and interpretation to inform action.

Continuous PHI training is available in two thirds of the responding institutions. However, many respondents are unaware of external training programs to expand their PHI skillset with the exception of Field Epidemiology Training Programs (FETPs). These have been previously identified as a prominent approach for strengthening the epidemic intelligence workforce [27]. All teams expressed interest in participating in PHI trainings, while a high proportion of teams reported to have the capability to provide such trainings to other institutions. Concepts for horizontal peer-to-peer training among PHI teams could provide viable avenues to match demand and offer of trainings. Challenges such as funding, time constraints, and lack of knowledge about available programs hinder participation in trainings. Addressing these barriers requires multilevel efforts from international, regional, and national institutions. Developing a standardized curriculum and guiding training frameworks in PHI would be a valuable asset for PHI teams. Institutions under the umbrella of the EIOS initiative (US CDC, RKI, WHO) are currently developing a PHI competency framework and curriculum [28].

Transitions experienced following the COVID-19 pandemic continue to shape the field of public health. There is considerable variability regarding the future of activities performed by the responding PHI teams. There is uncertainty concerning whether these activities will continue and to what extent, highlighting the susceptibility of PHI teams to external events. Key determinants to strengthen PHI and ensure the sustainability of activities were identified in the survey and include political commitment, long-term financing, and increased awareness of public health threats. At the health policy level, efforts are being intensified to prepare for future threats. Initiatives such as the Global Arbovirus Initiative [29] and the WHO’s pandemic accord [30] exemplify these efforts. On the implementation front, the use of One Health and all-hazards approaches are fundamental for early detection of public health threats and timely response. Possible strategies for PHI teams to achieve this include boosting the use of such approaches in routine practice [31] and intensifying cooperation with partners form other sectors, building trust to exchange information and data. While specific issues (e.g. nuclear risks, food safety) are often managed by specialized institutions, this data should be assessed in a holistic manner to guide decision making.

While the survey was distributed through several global networks, sampling bias cannot be excluded. A limitation to our study is the underrepresentation of some WHO regions (SEAR, WPR, AMR). This could be attributed, among other factors, to the limited reach of our sampling strategy, the absence of wide-reaching regional public health institutions that support with survey distribution in SEAR, WPR, AMR, or a potentially lower number of PHI teams active in those regions. Furthermore, the different understanding of partially overlapping definitions (e.g. PHI, EI, EBS) among participating organizations may have impacted the number of responses, depending on how institutions perceive and define their early warning and detection activities. This underscores the need for global common terminology achieved through updated guidance and advocacy.

In conclusion, our study reveals that further development of global PHI activities, methods and standards is needed. It can be achieved through global networking and capacity building, including training opportunities, horizontal peer-to-peer collaboration, and support from international institutions. Prioritizing these topics, securing funding and aligning actions would increase the robustness and sustainability of PHI systems, ultimately contributing to strengthening global health security.

Multidisciplinarity and cooperation are crucial to enhance effectiveness and avoid duplication of efforts. At the health policy level, comprehensive multisectoral approaches that cover surveillance for all relevant hazards must be ensured, along with strong political engagement and stable funding for sustainability. At the implementation level, there is potential for optimization through the uptake of more advanced methods, broader exchange, and more technical support. Integral strategies to foster PHI activities are crucial to strengthen global preparedness and response to public health threats.

## Supplementary Information

Supplementary Material 1.

## Data Availability

The datasets generated and analyzed during the current study are not publicly available due to the country names and agencies they contain. However, anonymized data could be made available from the corresponding author on reasonable request.

## Abbreviations

AFR: African Region
Africa CDC: Africa Centres for Disease Prevention and Control
AMR: Region of the Americas
EAR: Early Alerting and Reporting
EBS: Event-based surveillance
ECDC: European Centre for Disease Prevention and Control
EIOS: Epidemic Intelligence from Open Sources
EIOS: Epidemic intelligence
EMR: Eastern Mediterranean Region
EUR: European Region
EWRS: Early Warning and Response System
FETPs: Field Epidemiology Training Programs
GOARN: Global Outbreak Alert and Response Network
IANPHI: International Association of National Public Health Institutes
IBS: Indicator-based surveillance
IHR: International Health Regulations
NPHIs: National public health institutes
PHI: Public health intelligence
ProMed: Program for Monitoring Emerging Diseases
RKI: Robert Koch Institute
RPHIs: Regional public health institutions
SEAR: South-East Asian Region
US CDC: United States Centers for Disease Control and Prevention
WHO: World Health Organization
WPR: Western Pacific Region
