# Supplementary material for: Mapping the global public health intelligence landscape: a multiregional cross-sectional survey
Source: BMC Public Health. 2025 Dec 23;26:210. doi: 10.1186/s12889-025-25406-0 (PMC12805728; doi:10.1186/s12889-025-25406-0)

**Mapping the global Public Health Intelligence landscape: a multiregional cross-sectional survey**

**Supplementary material**

Table of contents

[**Table S1. List of the survey's questions and response options** 2](#_Toc208393787)

[**Figure S1. Duplicate analysis decision tree for identification of duplicated responses.** 6](#_Toc208393788)

[**Figure S2. Analysis flowchart for inclusion of responses detailing the exclusion criteria for responses, and number of responses excluded.** 7](#_Toc208393789)

[**Table S2. List of respondent countries included in the study.** 7](#_Toc208393790)

[**Table S3. Selected survey results by WHO Regions.** 8](#_Toc208393791)

[**Table S4. Public health intelligence data collection resources used by the identified PHI-teams by type of source, including a description of each tools and their access.** 9](#_Toc208393792)

[**Table S5. Selected survey results by type of institution** 11](#_Toc208393793)

[**Figure S3. Priority assessment of specific areas a) by WHO Region, and b) by type of institution.** 12](#_Toc208393794)

# **Table S1. List of the survey's questions and response options**

| **Nr.** | **Question** | **Response Options** |
| --- | --- | --- |
| Section 1: General information | | |
| 1 | In which country is your institution located? | Drop down menu |
| 2.1 | What kind of institution is it? | 1) National Public Health Institute  2) Regional Public Health Institute  3) Ministry of Health or equivalent 4) Other ministry 5) Academia  6) Other (specify) |
| 2.2 | If available, please share the website of your team/institution: | Free text |
| 3 | Does your team carry out surveillance activities for early detection of public health threats? These may include but not be limited to event-based surveillance, public health intelligence, epidemic intelligence, etc. | 1)Yes 2)No |
| 4 | What is the mandate of your team? Please choose all that apply. | 1) Inform policy makers 2) Inform the public 3Inform the scientific community 4) Generate scientific evidence for research purposes 5) Provide information for risk assessments 6) Inform action and response activities 7) Other |
| 5 | When did your team start its activities for early detection of public health threats? | 1) Before 2000 2) Between 2000 and 2009 3) Between 2010 and 2019 4) After 2019 |
| 6 | Did your team start its activities due to a specific health threat (e.g. after the emergence of a relevant pathogen or outbreak)?  If yes, please specify: | Free text |
| Section 2: Implementation of PHI activities | | |
| 7 | At which politico-administrative level does your team monitor events? Please choose all that apply. | 1) National level (own country) 2) Regional level (supranational, neighbouring countries) 3) International level (beyond neighbouring countries) |
| 8 | What kind of events does your team seek to detect? Please choose all that apply. | 1)Human health: communicable diseases 2) Human health: non-communicable diseases 3) Animal health 4) Environmental health 5) Food safety and security 6) Chemical hazards 7) Nuclear hazards 8) Healthcare-associated (nosocomial) infections and/or antimicrobial resistance 9) Health in natural disasters 10) Health in vulnerable population (e.g. refugees and/or displaced persons) 11) Other |
| 9 | Select the surveillance activities carried out by your team for early detection of public health threats. Please choose all that apply. | 1) Routine monitoring of established epidemiological indicators (e.g. incidence, prevalence, mortality) 2) Targeted social media scanning (e.g. Twitter, Facebook) 3) Targeted scanning of traditional media (e.g. newspapers, magazines, news agencies) 4) Community-based detection of relevant events 5) Facility-based detection of relevant events 6) Expert networks |
| 10 | Which methods for data collection does your team use for early detection of public health threats? Please choose all that apply. | 1) Newsletters or mailing lists  2) Official websites  3) ProMED  4) Outbreak News Today  5) GPHIN  6) EIOS  7) Healthmap  8) Medsys  9) Call centres/hotlines  10) SMS  11) E-mail  12) Other methods  13) Messaging apps (WhatsApp, Telegram, etc.) |
| 11 | Are there any specific events that you monitor on a regular basis due to their epidemiological importance (e.g. cholera, malaria, measles, influenza, HIV, antimicrobial resistance, healthcare-associated infections, undiagnosed deaths, mass-gatherings, haemorrhagic/jaundice syndomes, animal die-offs)? | 1) Yes. If yes, please specify:  2) No |
| 12 | What are your team’s early detection activities based on? Please note that this question refers solely to activities carried out for early threat detection, irrespective of other surveillance methods (e.g. conventional surveillance of specific diseases). Please move the slider to the most approriate position. | 1) Solely indicator-based surveillance 2) More indicator-based surveillance 3) Both indicator-based and event-based surveillance 4) More event-based surveillance 5) Solely event-based surveillance |
| 13 | How often does your team perform activities for early detection of public health threats on average? | 1) Daily (working days) 2) At least once a week but less than daily (working days) 3) Less than once a week 4) Event related only (seasonal or unexpected events) 5) Daily (including weekends and holidays) |
| 14 | Do you report the results of your team’s activities? Please choose all that apply. | 1) No 2) Yes, single pieces of information per e-mail or telephone 3) Yes, through regular reports shared internally within own institution 4) Yes, through regular reports shared with specific stakeholders 5) Yes, through regular reports shared externally and publicly accessible (e.g. on your institution’s website). If publicly available in a website, please provide the URL: |
| 15 | Do you upscale your early detection activities due to specific events (e.g. vector-borne diseases, mass gatherings, natural disasters)? Please choose all that apply. | 1) Yes, frequency of screening activities is increased 2) Yes, staff capacity is increased (more staff, more working hours) 3) No |
| 16 | Are you able to upscale your early detection activities on short notice? | 1) Yes 2) No |
| 17 | Does your team conduct more detailed assessments of events beyond detection and communication (e.g. risk assessments)? | 1) Yes, for all events 2) Yes, for selected events 3) No |
| 18 | For more detailed analyses, do you consult experts outside your team (e.g. other teams, subject matter experts, other specialized institutions)? | 1) Yes 2) No |
| 19 | Do you cooperate with other teams conducting early detection activities outside of your institution? | 1) Yes2) No |
| Section 3: Human resources and training | | |
| 20 | Does your institution have dedicated human resources for early detection activities only? | 1) Yes, permanently available 2) Yes, available only for selected events 3) No, staff is pooled from other teams or outsourced when needed |
| 21 | Are there specific requirements for staff joining your team? Please choose all that apply. | 1) No 2) Yes, a university degree in a relevant field 3) Yes, other professional qualifications  4) Yes, completion of an internal course or training in early detection of public health threats 5) Yes, completion of an external course or training in early detection of public health threats 6) Yes, proof of previous work experience in early detection of public health threats 7) Other. If other, please specify: |
| 22 | What are the professional backgrounds of your current team’s staff? Please choose all that apply. | 1) Medicine 2) Nursing 3) Veterinary medicine 4) Epidemiology 5) Public health 6) Biology 7) Statistics 8) Geography 9) Sociology 10) Anthropology 11) IT 12) Other. If other, please specify: |
| 23 | Does your institution offer continuous training on early detection of public health threats for the team’s staff? Please choose all that apply. | 1) Yes, continued training is offered and mandatory for staff 2) Yes, continued training is offered and optional for staff 3) No |
| 24 | Do you know any external training programs for early detection of public health threats in your country or internationally? | 1) Yes. If yes, please specify:  2) No |
| 25 | Have you participated in an external training program for early detection of public health threats? Please choose all that apply. | 1) Yes, in a national program 2) Yes, in an international program 3) No |
| 26 | Would your team be willing to receive trainings for early detection of public health threats? | 1) Yes 2) No |
| 27 | Does your team currently offer training activities for other teams performing early detection activities of public health threats? | 1) Yes 2) No |
| 28.a | Would your team be able to scale up your current training activities for further teams performing early detection activities of public health threats? | 1) Yes 2) No |
| 28.b | Would your team be able to offer training activities for other teams performing early detection activities of public health threats? | 1) Yes 2) No |
| Section 4: Perception and opinion questions | | |
| 29 | From 1 to 4 how would you prioritise the needs of your team in each of the following categories? | 1) Training and method optimization  2) Software and digital solutions  3) Equipment, including hardware and infrastructure  4) Human resources/staff  5) Networking and knowledge exchange |
| 30 | Which opportunities can you identify to strengthen your team and help enhance its activities? Here, opportunities are considered to be favourable situations external to your team (e.g. increased awareness for public health threats, political will, increased funding). | Free text |
| 31 | Which challenges does your team face? Here, challenges are considered to be unfavourable external to your team (e.g. lacking awareness for public health threats, lacking political will, reduced funding). | Free text |
| Other questions | | |
| 32 | The emergence of the COVID-19 pandemic had a significant impact on the public health activities worldwide. With the pandemic reaching a transition point, do you expect any changes in your team’s activities in the near future? | 1) No, activities will continue as they are 2) Yes, activities will be scaled down 3) Yes, activities will be scaled up 4) No/I don’t know |
| 33 | Are you part of a network and/or community of practice for exchange with teams or units in other institutions carrying out similar early detection surveillance activities? | 1) Yes. If yes, please specify which networks and/or communities of practice:  2) No |
| 34 | Is your team interested in connecting with other teams responding to this survey? | 1) Yes. If yes, what would be your expectations of such a network?  2) No |
| 35 | How did you receive this survey? Please choose all that apply. | 1) International Association of National Public Health Institutes (IANPHI) 2) WHO Hub for Pandemic and Epidemic Preparedness (EIOS community) 3) Africa Centres for Disease Control and Prevention (Africa CDC) 4) European Centre for Disease PRevention and Control (ECDC) 5) US Centers for Disease Control and prevention (US CDC) 6) Robert Koch Institute (RKI) 7) Other colleagues within own institution 8) Other |
| 36 | Please add any additional information or comments you would like to share: | Free text |

# **Figure S1. Duplicate analysis decision tree for identification of duplicated responses.**


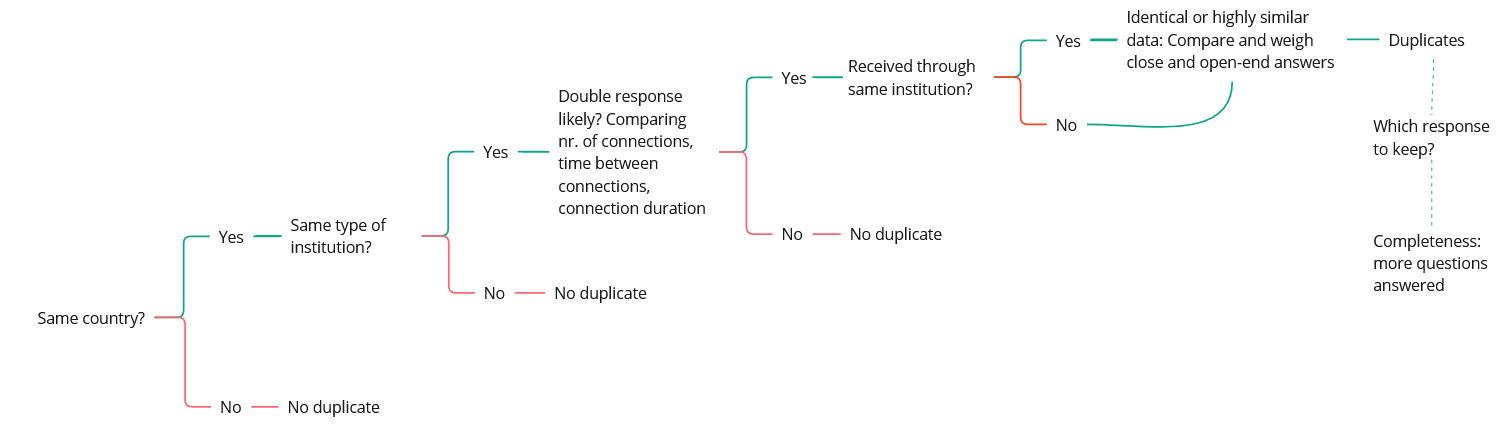


# **Figure S2. Analysis flowchart for inclusion of responses detailing the exclusion criteria for responses, and number of responses excluded.**

**
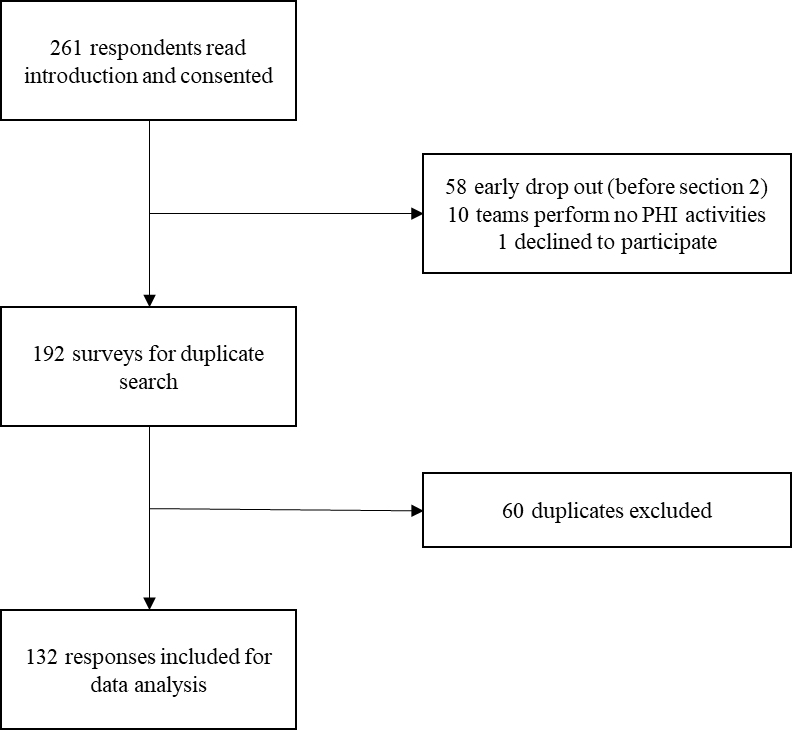
**

# **Table S2. List of respondent countries included in the study.**

# **Table S3. Selected survey results by WHO Regions.**

|  | **AFR** | **AMR** | **EMR** | **EUR** | **WPR/SEAR** | **Total** |
| --- | --- | --- | --- | --- | --- | --- |
| **Type of institution** | | | | | |  |
| Academia | 4 (7%) | 0 (0%) | 1 (12%) | 0 (0%) | 0 (0%) | **5** |
| Ministry of Health | 16 (27%) | 3 (38%) | 0 (0%) | 6 (13%) | 4 (36%) | **29** |
| NGO | 8 (13%) | 0 (0%) | 0 (0%) | 0 (0%) | 0 (0%) | **8** |
| National Public Health Institute | 14 (23%) | 5 (62%) | 4 (50%) | 25 (56%) | 5 (45%) | **53** |
| Other | 6 (10%) | 0 (0%) | 1 (12%) | 4 (9%) | 1 (9%) | **12** |
| Other ministry | 2 (3%) | 0 (0%) | 1 (12%) | 3 (7%) | 0 (0%) | **6** |
| Regional Public Health Institute | 5 (8%) | 0 (0%) | 1 (12%) | 1 (2%) | 1 (9%) | **7** |
| United Nations | 5 (8%) | 0 (0%) | 0 (0%) | 6 (13%) | 0 (0%) | **11** |
| **Total** | **60** | **8** | **8** | **45** | **11** | **132** |
| **Mandates** | | | | | |  |
| Inform policy makers | 45 (75%) | 7 (88%) | 6 (75%) | 40 (89%) | 11 (100%) | **109** |
| Inform the public | 39 (65%) | 6 (75%) | 5 (62%) | 31 (69%) | 11 (100%) | **92** |
| Inform the scientific community | 42 (70%) | 5 (62%) | 4 (50%) | 30 (67%) | 8 (73%) | **89** |
| Generate scientific evidence for research | 35 (58%) | 4 (50%) | 4 (50%) | 18 (40%) | 6 (55%) | **67** |
| Provide information for risk assessments | 47 (78%) | 6 (75%) | 6 (75%) | 41 (91%) | 11 (100%) | **111** |
| Inform action and response activities | 53 (88%) | 8 (100%) | 6 (75%) | 42 (93%) | 11 (100%) | **120** |
| Other | 9 (15%) | 1 (12%) | 4 (50%) | 7 (16%) | 0 (0%) | **21** |
| **Total** | **60** | **8** | **8** | **45** | **11** | **132** |
| **Surveillance activities** | | | | | |  |
| Routine monitoring of established epi indicators | 59 (98%) | 5 (62%) | 5 (62%) | 41 (91%) | 10 (91%) | **120** |
| Targeted social media scanning | 35 (58%) | 3 (38%) | 6 (75%) | 21 (47%) | 5 (45%) | **70** |
| Targeted screening of traditional media | 36 (60%) | 5 (62%) | 7 (88%) | 32 (71%) | 8 (73%) | **88** |
| Community-based detection of relevant events | 47 (78%) | 4 (50%) | 6 (75%) | 21 (47%) | 6 (55%) | **84** |
| Facility-based detection of relevant events | 49 (82%) | 5 (62%) | 5 (62%) | 23 (51%) | 6 (55%) | **88** |
| Expert networks | 22 (37%) | 4 (50%) | 4 (50%) | 35 (78%) | 7 (64%) | **72** |
| **Total** | **60** | **8** | **8** | **45** | **11** | **132** |
| **Monitored hazards** | | | | | |  |
| Human health: communicable diseases | 57 (95%) | 8 (100%) | 6 (75%) | 44 (98%) | 10 (91%) | **125** |
| Healthcare-associated infections antimicrobial resistance | 26 (43%) | 5 (62%) | 3 (37%) | 17 (38%) | 4 (36%) | **87** |
| Health in natural disasters | 41 (68%) | 5 (62%) | 6 (75%) | 16 (36%) | 5 (45%) | **85** |
| Food safety and security | 41 (68%) | 5 (62%) | 5 (62%) | 20 (44%) | 7 (64%) | **81** |
| Environmental health | 40 (67%) | 4 (50%) | 5 (62%) | 25 (56%) | 7 (64%) | **78** |
| Health in vulnerable populations | 31 (52%) | 4 (50%) | 5 (62%) | 18 (40%) | 3 (27%) | **76** |
| Animal health | 21 (35%) | 4 (50%) | 4 (50%) | 11 (24%) | 2 (18%) | **73** |
| Chemical hazards | 32 (53%) | 6 (75%) | 5 (62%) | 34 (76%) | 10 (91%) | **61** |
| Human health: non-communicable diseases | 39 (65%) | 4 (50%) | 6 (75%) | 28 (62%) | 8 (73%) | **55** |
| Nuclear hazards | 35 (58%) | 4 (50%) | 5 (62%) | 27 (60%) | 5 (45%) | **42** |
| Other | 4 (7%) | 2 (25%) | 1 (12%) | 3 (7%) | 1 (9%) | **11** |
| **Total** | **60** | **8** | **8** | **45** | **11** | **132** |
| **Capacity for scaling up surveillance activities** | | | | | |  |
| No | 9 (16%) | 1 (12%) | 2 (29%) | 11 (25%) | 1 (10%) | **24** |
| Yes | 48 (84%) | 7 (88%) | 5 (71%) | 33 (75%) | 9 (90%) | **102** |
| **Total** | **57** | **8** | **7** | **44** | **10** | **126** |
| **Detailed assessment of events** | | | | | |  |
| No | 5 (9%) | 1 (12%) | 1 (17%) | 4 (9%) | 0 (0%) | **11** |
| Yes, for all events | 22 (39%) | 4 (50%) | 2 (33%) | 5 (11%) | 3 (30%) | **36** |
| Yes, for selected events | 29 (52%) | 3 (38%) | 3 (50%) | 35 (80%) | 7 (70%) | **77** |
| **Total** | **56** | **8** | **6** | **44** | **10** | **124** |
| **Dedicated human resources for PHI** | | | | | |  |
| No, staff is pooled from other teams/outsourced when needed | 16 (30%) | 2 (25%) | 1 (17%) | 13 (30%) | 2 (20%) | **34** |
| Yes, available only for selected events | 9 (17%) | 1 (12%) | 1 (17%) | 6 (14%) | 3 (30%) | **20** |
| Yes, permanently available | 29 (54%) | 5 (62%) | 4 (67%) | 25 (57%) | 5 (50%) | **68** |
| **Total** | **54** | **8** | **6** | **44** | **10** | **122** |
| **Offering of training activities** | | | | | |  |
| No | 19 (36%) | 3 (38%) | 1 (17%) | 21 (48%) | 5 (56%) | **49** |
| Yes | 34 (64%) | 5 (62%) | 5 (83%) | 23 (52%) | 4 (44%) | **71** |
| **Total** | **53** | **8** | **6** | **44** | **9** | **120** |

# **Table S4. Public health intelligence data collection resources used by the identified PHI-teams by type of source, including a description of each tools and their access.**

| **Type of source** | **Name** | **Description** | **Access** |
| --- | --- | --- | --- |
| **Platforms** (allow for exchange among users) | *Epidemic Intelligence from Open Sources (*EIOS*)* | System developed by WHO and the European Commission to scan publicly available sources of information | Restricted to members, no fee required |
|  | WHO *Event Information Site (*WHO EIS*)* | Platform for exchange information sharing with IHR national focal points | Restricted to members, no fee required |
|  | *EU Early Warning and Response System (*EU EWRS) | Tool for monitoring public health threats in the EU. Accessed limited to ECDC, EU Member States and the Directorate General Health and Food Safety (SANTE). | Restricted to members, no fee required |
|  | EU EpiPulse | Online portal for European public health authorities and partner organisations for threat detection, monitoring, assessment and outbreak through exchange of data on infectious diseases. | Restricted to members, no fee required |
|  | *Global Public Health Intelligence Network* (GPHIN) | Network set up by the Government of Canada in collaboration with the World Health Organization. It encompasses an  automated web-based system to scan media reports. | Restricted to members, fee required |
|  | *Global Infectious Disease and Epidemiology Network (*GIDEON) | Dynamic reference database containing information on tropical and infectious diseases, epidemiology and microbiology. | Restricted to members, fee required |
|  | *Medical Information System (*Medsys*)* | Media monitoring system for rapid detection of potential public health threats scanning information from media reports. | Restricted to members, fee required |
| **Websites** (do not allow for exchange among users) | *Program for Monitoring Emerging Diseases (*ProMed) | System developed by the International Society for Infectious Diseases (ISID) that reports infectious disease outbreaks at a global level. Reports are produced and commentary provided by expert moderators. | Freely accessible, no fee required |
|  | HealthMap | Machine learning tool for tracking infectious disease outbreaks on a global level by aggregating informal online sources, including news media and social media posts. | Freely accessible, no fee required |
|  | ReliefWeb | ReliefWeb is a humanitarian information service provided by the United Nations Office for the Coordination of Humanitarian Affairs (OCHA).  Content from different institutions and media is monitored, collected and curated and delivered through a website. | Freely accessible, no fee required |
| **Magazines/ blogs** | Outbreak News Today | News website focused on infectious diseases, also covering non- communicable diseases and other health topics with a global focus. | Freely accessible, no fee required |
|  | FluTrackers | Online forum for volunteer sharing of information on disease and human rights. | Freely accessible, no fee required |
|  | CIDRAP  *Center for Infectious Disease Research and Policy* | News portal providing articles on infectious diseases and further public health-relevant topics | Freely accessible, no fee required |
|  | Avian Flu Diary | Personal blog with current contents on influenza, emerging infectious diseases and further public health-relevant topics. | Freely accessible, no fee required |
|  | Croftsblog | Personal blog with current contents on influenza, emerging infectious diseases and further public health-relevant topics. | Freely accessible, no fee required |
| **Automatic web aggregators** | Google Alerts | Service that notifies the user about changes in specific online contents. | Freely accessible |
|  | Feedly | Application for web browsers that tracks and compiles news from different sources. | Account required, fee required for some features |
|  | Factiva | Platform compiling content from news, data and information sources with a global scope. | Account required, fee required |
| **Periodic reports** | WHO Diseases Outbreak News reports | Reports on confirmed acute public health events or potential events of concern. | Freely accessible, no fee required |
|  | ECDC Communicable Diseases Threat Report | Weekly reports summarizing information collected through epidemic intelligence activities on infectious disease events relevant to the EU. | Version freely available, no fee required; version restricted to users, no fee required |
|  | Africa CDC situation reports | Weekly reports with timely updates on public health events of concern in Africa. | Freely accessible, no fee required |

| **Table S5. Selected survey results by type of institution** | | | |  |  |  |  |  |  |
| --- | --- | --- | --- | --- | --- | --- | --- | --- | --- |
|  | **Academia** | **MoH** | **NGO** | **NPHI** | **Other** | **Other ministry** | **RPHI** | **UN** | **Total** |
| **Mandate** | | | | | | | | |  |
| Inform policy makers | 2 (40%) | 26 (90%) | 4 (50%) | 51 (96%) | 10 (83%) | 4 (67%) | 4 (50%) | 8 (73%) | **109** |
| Inform the public | 1 (20%) | 27 (93%) | 5 (62%) | 39 (74%) | 8 (67%) | 3 (50%) | 3 (38%) | 6 (55%) | **92** |
| Inform the scientific community | 2 (40%) | 23 (79%) | 4 (50%) | 38 (72%) | 7 (58%) | 3 (50%) | 4 (50%) | 8 (73%) | **89** |
| Generate scientific evidence for research purposes | 3 (60%) | 10 (34%) | 3 (38%) | 35 (66%) | 7 (58%) | 1 (17%) | 3 (38%) | 5 (45%) | **67** |
| Provide information for risk assessments | 1 (20%) | 28 (97%) | 6 (75%) | 48 (91%) | 11 (92%) | 4 (67%) | 5 (62%) | 8 (73%) | **111** |
| Inform action and response activities | 2 (40%) | 29 (100%) | 7 (88%) | 52 (98%) | 12 (100%) | 4 (67%) | 6 (75%) | 8 (73%) | **120** |
| Other | 2 (40%) | 1 (3%) | 1 (12%) | 10 (19%) | 3 (25%) | 1 (17%) | 1 (12%) | 2 (18%) | **21** |
| **Total** | **5** | **29** | **8** | **53** | **12** | **6** | **8** | **11** | **132** |
| **Surveillance activities** | | | | | | | | |  |
| Routine monitoring of established epi indicators | 4 (80%) | 27 (93%) | 8 (100%) | 49 (92%) | 11 (92%) | 5 (83%) | 6 (75%) | 10 (91%) | **120** |
| Targeted social media scanning | 1 (20%) | 15 (52%) | 5 (62%) | 28 (53%) | 6 (50%) | 2 (33%) | 6 (75%) | 7 (64%) | **70** |
| Targeted screening of traditional media | 1 (20%) | 18 (62%) | 5 (62%) | 35 (66%) | 8 (67%) | 5 (83%) | 7 (88%) | 9 (82%) | **88** |
| Community-based detection of relevant events | 2 (40%) | 20 (69%) | 7 (88%) | 31 (58%) | 8 (67%) | 2 (33%) | 6 (75%) | 8 (73%) | **84** |
| Facility-based detection of relevant events | 3 (60%) | 20 (69%) | 6 (75%) | 35 (66%) | 8 (67%) | 3 (50%) | 5 (62%) | 8 (73%) | **88** |
| Expert networks | 1 (20%) | 13 (45%) | 3 (38%) | 34 (64%) | 5 (42%) | 2 (33%) | 5 (62%) | 9 (82%) | **72** |
| **Total** | **5** | **29** | **8** | **53** | **12** | **6** | **8** | **11** | **132** |
| **Capacity for scaling up** | | | | | | | | |  |
| No | 3 (75%) | 4 (14%) | 2 (25%) | 13 (25%) | 1 (10%) | 1 (20%) | 0 (0%) | 0 (0%) | **24** |
| Yes | 1 (25%) | 25 (86%) | 6 (75%) | 40 (75%) | 9 (90%) | 4 (80%) | 7 (100%) | 10 (100%) | **102** |
| **Total** | **4** | **29** | **8** | **53** | **10** | **5** | **7** | **10** | **126** |
| **Detailed assessment of events** | | | | | | | | |  |
| No | 1 (25%) | 2 (7%) | 1 (12%) | 3 (6%) | 2 (20%) | 1 (25%) | 1 (14%) | 0 (0%) | **11** |
| Yes, for all events | 1 (25%) | 10 (34%) | 4 (50%) | 10 (19%) | 4 (40%) | 0 (0%) | 4 (57%) | 3 (30%) | **36** |
| Yes, for selected events | 2 (50%) | 17 (59%) | 3 (38%) | 39 (75%) | 4 (40%) | 3 (75%) | 2 (29%) | 7 (70%) | **77** |
| **Total** | **4** | **29** | **8** | **52** | **10** | **4** | **7** | **10** | **124** |
| **Dedicated human ressources for PHI** | | | | | | | | |  |
| No, staff is pooled from other teams/outsourced when needed | 2 (50%) | 10 (34%) | 1 (12%) | 16 (31%) | 1 (10%) | 0 (0%) | 3 (43%) | 1 (12%) | **34** |
| Yes, available only for selected events | 1 (25%) | 4 (14%) | 1 (12%) | 13 (25%) | 1 (10%) | 0 (0%) | 0 (0%) | 0 (0%) | **20** |
| Yes, permanently available | 1 (25%) | 15 (52%) | 6 (75%) | 23 (44%) | 8 (80%) | 4 (100%) | 4 (57%) | 7 (88%) | **68** |
| **Total** | **4** | **29** | **8** | **52** | **10** | **4** | **7** | **8** | **122** |
| **Offering of training activities** | | | | | | | | |  |
| No | 2 (50%) | 15 (52%) | 4 (50%) | 21 (42%) | 3 (30%) | 3 (75%) | 0 (0%) | 1 (12%) | **49** |
| Yes | 2 (50%) | 14 (48%) | 4 (50%) | 29 (58%) | 7 (70%) | 1 (25%) | 7 (100%) | 7 (88%) | **71** |
| **Total** | **4** | **29** | **8** | **50** | **10** | **4** | **7** | **8** | **120** |

# **Figure S3. Priority assessment of specific areas a) by WHO Region, and b) by type of institution.**

**a)**


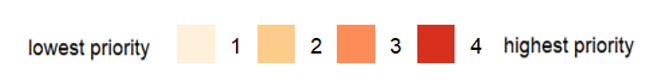

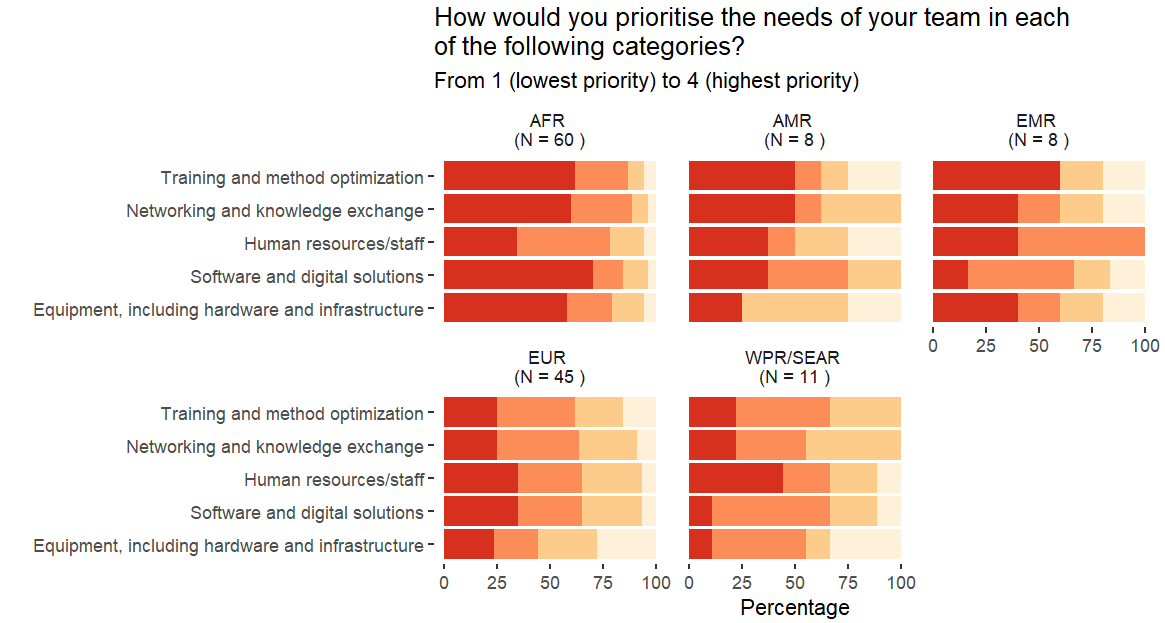


**b)**


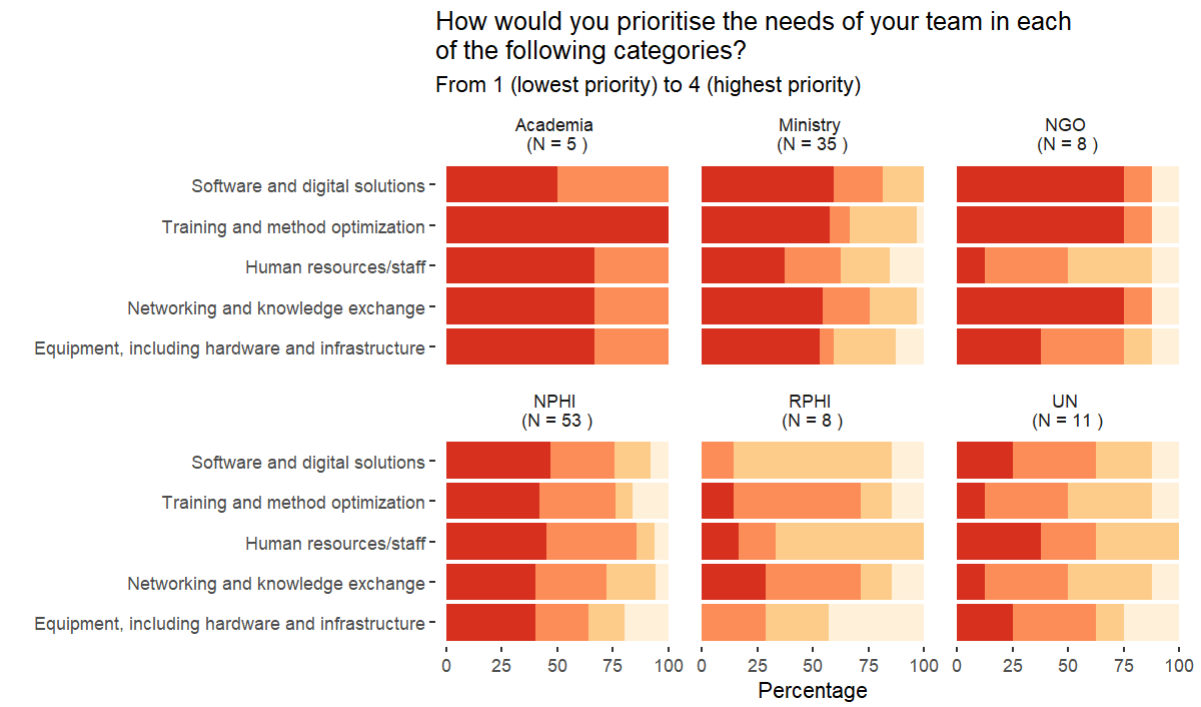

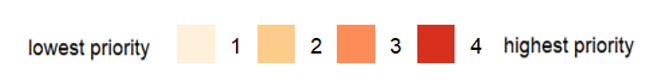

Supplement: Supplementary file 1 — Supplementary Material 1. [file 12889_2025_25406_MOESM1_ESM.docx]
